# Evidence that endpoint feedback facilitates intermanual transfer of visuomotor force learning by a cognitive strategy

**DOI:** 10.1152/jn.00008.2021

**Published:** 2021-12-09

**Authors:** Jack De Havas, Patrick Haggard, Hiroaki Gomi, Sven Bestmann, Yuji Ikegaya, Nobuhiro Hagura

**Affiliations:** ^1^NTT Communication Science Laboratories, Atsugi, Japan; ^2^Institute of Cognitive Neuroscience, University College London, London, United Kingdom; ^3^Center for Information and Neural Networks, National Institute for Information and Communications Technology, Osaka, Japan; ^4^UCL Queen Square Institute of Neurology, Department of Clinical and Movement Neurosciences, University College London, London, United Kingdom; ^5^Wellcome Centre for Human Neuroimaging, University College London, London, United Kingdom; ^6^Faculty of Pharmaceutical Sciences, Graduate School of Pharmaceutical Sciences, The University of Tokyo, Tokyo, Japan; ^7^Graduate School of Frontier Biosciences, Osaka University, Osaka, Japan

**Keywords:** cognitive strategy, intermanual transfer, visual feedback, visuomotor adaptation, visuomotor gain

## Abstract

Humans continuously adapt their movement to a novel environment by recalibrating their sensorimotor system. Recent evidence, however, shows that explicit planning to compensate for external changes, i.e., a cognitive strategy, can also aid performance. If such a strategy is planned in external space, it should improve performance in an effector-independent manner. We tested this hypothesis by examining whether promoting a cognitive strategy during a visual-force adaptation task performed in one hand can facilitate learning for the opposite hand. Participants rapidly adjusted the height of visual bar on screen to a target level by isometrically exerting force on a handle using their right hand. Visuomotor gain increased during the task and participants learned the increased gain. Visual feedback was continuously provided for one group, whereas for another group only the endpoint of the force trajectory was presented. The latter has been reported to promote cognitive strategy use. We found that endpoint feedback produced stronger intermanual transfer of learning and slower response times than continuous feedback. In a separate experiment, we found evidence that aftereffects are reduced when only endpoint feedback is provided, a finding that has been consistently observed when cognitive strategies are used. The results suggest that intermanual transfer can be facilitated by a cognitive strategy. This indicates that the behavioral observation of intermanual transfer can be achieved either by forming an effector-independent motor representation or by sharing an effector-independent cognitive strategy between the hands.

**NEW & NOTEWORTHY** The causes and consequences of cognitive strategy use are poorly understood. We tested whether a visuomotor task learned in a manner that may promote cognitive strategy use causes greater generalization across effectors. Visual feedback was manipulated to promote cognitive strategy use. Learning consistent with cognitive strategy use for one hand transferred to the unlearned hand. Our result suggests that intermanual transfer can result from a common cognitive strategy used to control both hands.

## Graphical abstract

**Figure:**
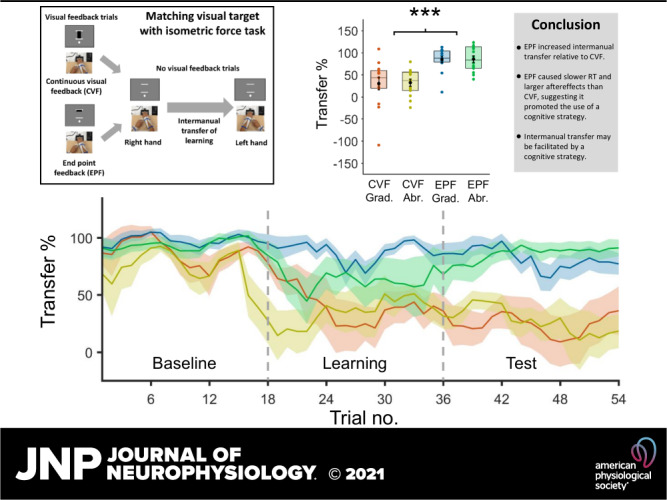

## INTRODUCTION

When hitting a tennis ball on a windy day, you might aim slightly to the side of where you want the ball to land to take the direction of the wind into account. As such, humans can explicitly shift the aim of their actions to compensate for external perturbations, known as a cognitive strategy. Although error-based motor learning has traditionally been considered a single implicit sensorimotor recalibration process (1), recent work has described the contribution of such cognitive strategies to motor learning (2, 3).

Cognitive strategies differ from motor adaptation in terms of how and where in the brain they are implemented (4, 5). They also likely differ in terms of how sensory feedback is processed, with cognitive strategies producing learning that weights performance error above sensory prediction error to a greater extent than learning by adaptation (6). Learning using cognitive strategies and motor adaptation overlap throughout sensorimotor tasks (7) but can be separated by manipulating task instructions (8, 9). In this study, we investigate how cognitive strategy use generalizes across effectors in motor learning, by examining the intermanual transfer of motor learning.

How motor adaptation tasks learned on one hand transfer to the other has been extensively studied (10–13). This intermanual transfer has been traditionally ascribed to motor adaptation happening in each hemisphere (14); however, whether a cognitive strategy can facilitate intermanual transfer is still under debate. Some studies have reported that the use of a cognitive strategy during motor adaptation tasks can facilitate intermanual transfer (15, 16), whereas others have not (17–19). In these studies, cognitive strategy use has been promoted by introducing an abrupt change in the perturbation (i.e., sudden introduction of the visuomotor rotation or force field), purposefully making the participants aware of the perturbation. However, this method may potentially induce interindividual variability in cognitive strategy use, depending on the size of the change and differences in individual sensitivity to that change (16).

In this study, we promote the use of cognitive strategy during motor learning by showing only the end point of the action (endpoint feedback; EPF), as opposed to showing the feedback continuously throughout the action (continuous visual feedback; CVF). CVF provides both visual sensory prediction errors and visual performance errors relating to the entire action. EPF conversely involves a single visual performance error signal pertaining to goal completion. As cognitive strategies may preferentially weight performance error, we predict that restricting visual feedback to a salient performance error signal may shift the means of task learning away from motor adaptation and toward strategy use. Indeed, aftereffects upon the removal of a visuomotor perturbation, a hallmark of motor adaptation, are attenuated by EPF relative to CVF (20–22).

In the task, participants isometrically and ballistically exerted force on a gripped handle to control a visual bar height on screen to reach a target height. After a baseline phase, the visuomotor gain (force to bar-height transformation) increased, requiring participants to modify their motor command to maintain performance. A 2 × 2 across-subjects factorial design was used for the first experiment, with factors of visual feedback (EPF vs. CVF) and perturbation schedule (abrupt vs. gradual increase of visuomotor gain), conceptually mimicking previous studies (16, 17–19).

We first assessed whether EPF and abrupt gain change promoted cognitive strategies by examining reaction times. Verbal instructions to use cognitive strategies, tasks showing only EPF, and tasks where perturbations are changed abruptly, all exhibit slow response times (23, 24). In addition, limiting response times reduces strategic learning, as evidenced by increased aftereffects (25). Thus, if EPF and abrupt gain change do promote strategy use they should be associated with prolonged reaction times (RT). Second, we examined the transfer rate of a gain change learned with the right hand to the left hand. As planning based on performance error is computed in target space (26), e.g., a strategy to aim right or left of where the target appears to be located, such strategies should be applicable for controlling either hand. Thus, cognitive strategy use should facilitate intermanual transfer. Finally, in a separate experiment, we provided independent evidence that the type of visual feedback provided in our current force production task can indeed promote strategy use, by showing that this factor may influence the size of aftereffects, consistent with previous reports (23, 25, 27).

## MATERIALS AND METHODS

### Equipment

**Figure 1.**
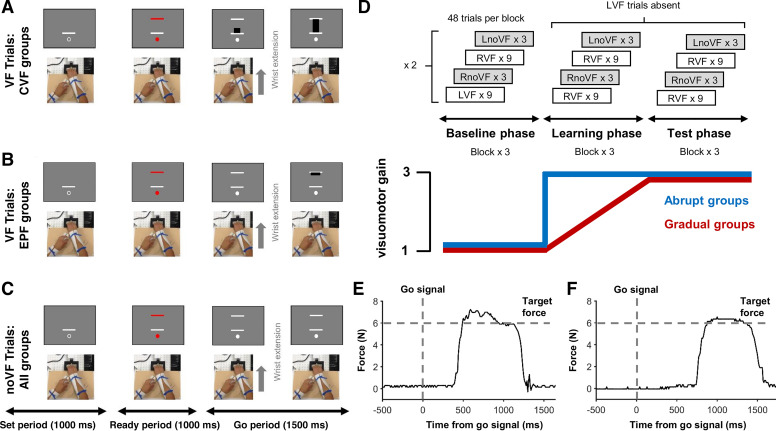
Task structure and single-trial results. *A*: visual feedback (VF) trials in continuous visual feedback (CVF) groups, where isometric wrist force was continuously shown on screen as the height of a black bar. *B*: for endpoint feedback (EPF) groups, during visual feedback trials force was displayed as a static black bar, which appeared once the wrist extension was completed. *C*: “No visual feedback trials” (noVF) were identical for all groups and involved participants making wrist extensions of appropriate strength in the absence of any visual feedback. *D*: the experiment had a baseline, learning, and test phase, each with 3 blocks of 48 trials. In the baseline phase, participants alternated between sets of 9 “visual feedback trials” and 3 “no visual feedback trials,” using either the right or left hand (pseudorandomized). During the learning phase, visuomotor gain increased from 1 to 3, either abruptly (abrupt gain change groups), or via linear increments across visual feedback trials (gradual gain change groups). Left hand visual feedback (LVF) trials were absent during the learning and test phase, meaning that the gain change was only experienced directly when using the right hand. *E*: a single representative right hand trial from a participant in one of the CVF groups, showing force increase toward the visual target in response to the go signal. *F*: a representative right hand trial from an EPF group participant, showing force increase toward the visual target in response to the go signal. LnoVF and RnoVF, no visual feedback trials with the left and right hand, respectively; RVF, right hand visual feedback.

Participants were seated and held a plastic handle (aligned to midline, navel height) in a power grip. The handle was instrumented with force sensors, which consisted of an optical strain gauge composed of a digital fiber sensor (FS-N10; Keyence corp.) and a limited-reflective fiber unit (FU-38; Keyence corp.) (28). Participants’ arms were pronated and attached to custom-built forearm restraints, which consisted of moulded plastic with Velcro straps at either end (Fig. 1). The restraints slotted into adjustable runners attached to a solid wooden board, which allowed rapid arm switching during the task. The force data from the handle were processed online by the connected PC for online presentation of the force (sample rate = 100 Hz). Experimental stimuli were created using MATLAB (2017) with Psychophysics Toolbox extensions (29, 30) and were presented via a flat screen monitor (27 in. LCD, 1,440 × 900 pixels resolution pixels, 60 Hz refresh rate) positioned 40 cm in front of participants.

### Participants

A total of 58 people participated in *experiment 1*, of which two were excluded for failing to comply with the task, leaving 14 participants per group. CVF Abrupt (Abr): *n* = 14, females = 7 [age mean (Mn) = 23.8, SD = 4.8]. CVF Gradual (Grd): *n* = 14, females = 6, (age Mn = 25, SD = 5.6). EPF Abr: *n* = 14, females = 5 (age Mn = 23.4, SD = 3). EPF Grd: *n* = 14, females = 5 (age Mn = 24.4, SD = 6.3).

A total of 33 people participated in *experiment 2*. Four participants were excluded from the analysis, two of which were due to mechanical issues and two of which were due to a failure to comply with task instructions. This left 14 participants in the CVF group (females = 8, age Mn = 22.6, SD = 1.5) and 15 participants in the EPF group (females = 7, age Mn = 21.3, SD = 1.8), none of whom had participated in *experiment 1*.

Both experiments were undertaken with the understanding and written consent of each participant in accordance with the Code of Ethics of the World Medical Association (Declaration of Helsinki) and with approval of the NICT ethical committee. No adverse events occurred during either experiment.

### Procedure

#### Experiment 1.

The task was to control the level of force exerted on a handle to reach a target level. The height of the bar on the monitor served as the level of exerted force, and in each trial, participants were asked to set the height of the bar to the target level by isometrically and ballistically exerting force on the handle. The task started with the baseline phase, followed by a learning phase and then the test phase. After the baseline phase, participants had to adapt to a three times increase in visuomotor gain in the learning phase (i.e., the same amount of force applied to the handle during the baseline would produce three times as much bar height on screen). The increased gain remained stable during the test phase.

Each trial began with participants viewing a white open circle positioned under a white line while holding the handle in a relaxed position (Fig. 1, *A–C*). The circle served as a fixation point and the white bar indicated the baseline force level; the force level when the participants did not intentionally exert force on the handle. After 1,000 ms, the fixation circle was filled with red and a red target force line appeared at one of three equispaced locations above the baseline. Each line height corresponded to three different force levels (3, 6, 9 N during baseline phase, 1, 2, 3 N during test phase). Participants prepared their response (1,000 ms) until the central fixation circle and the target force line turned from red to white (Go signal). In response to the Go signal, participants executed isometric wrist extensor contractions of appropriate strength as quickly as possible.

The experiment was designed as a 2 (visual feedback type) × 2 (perturbation schedule) between-subject factorial design, where four different combination of factors were assigned to four different groups of participants.

For the factor of visual feedback type, in one condition, the amount of vertical force exerted on the handle was continuously presented on screen as the height of a solid black bar (continuous visual feedback; CVF) (Fig. 1*A*). In the other condition, feedback was provided instead via a solid black line indicating the force level at the point in time when force velocity had reached its peak (end point feedback; EPF) (Fig. 1*B*). For the factor of perturbation schedule, in one condition, the visuomotor gain increased by three times abruptly at the first trial of the learning phase (Abr). In the other condition, the gain increased gradually (linearly) over the course of learning phase (Grd).

The experiment began with 4 practice blocks (2 blocks per hand) of 48 trials with visual feedback (VF). The baseline phase (Fig. 1*D*) consisted of 3 blocks of 48 trials. Each block consisted of four iterations of nine VF trials (3 × low, medium, and high force targets, randomized) followed by three trials without visual feedback of the exerted force level (noVF) (1 × low, medium, and high force targets, randomized). Two of the four sets of nine VF trials used the right hand (RVF) and two used the left hand (LVF). Likewise, two of the four sets of three noVF trials used the right hand (RnoVF) and two used the left hand (LnoVF). In total, there were 54 RVF trials, 54 LVF trials, 36 RnoVF trials, and 36 LnoVF trials in the baseline phase. Hand order within each block was randomized.

Learning and test phase each had three blocks of 48 trials, consisting of similar types of trials as the baseline phase. However, the LVF trials were replaced with the RVF trials, thus, both phases had a total of 108 RVF trials, 36 RnoVF trials, and 36 LnoVF trials. This was to prevent any visual error-based learning from occurring for left hand trials, whereas the right hand adapted to the change in the visuomotor gain. Therefore, any visual gain learning observed in the LnoVF trials could be attributed to learning transferred from the right hand. Participants had a 2-min rest after every task block. The experiment lasted 1.5 h.

#### Experiment 2.

The goal of *experiment 2* was to establish whether EPF produced smaller aftereffects than CVF in our force production task, as previous literature using reaching movements suggested that strategy use causes reduced aftereffects. The force generation task in *experiment 2* was the same as *experiment 1*. Once again participants exerted force on the handle to reach the same visual targets on screen. But here participants only used their right hand to respond throughout the experiment and gain changes were identical across the two visual feedback groups (Fig. 3*A*). After two practice blocks, participants performed two blocks (45 trials per block) with a visuomotor gain of 3 (baseline phase). Thus, in total, they experienced 180 trials with the initial gain of 3. This was followed by two blocks in which the gain gradually decreased to one. Then followed four blocks in which the gain was fixed at 1. The final two blocks with gain fixed to 1 was defined as the test phase, which was used to assess how well participants had learned the gain change. All trials up to this point only included visual feedback trials.

After these blocks of learning the decreased gain from the baseline, on the 10th trial of *block 9*, the gain suddenly changed back to 3. After this sudden gain change, a noVF trial was presented on every third trial for the remainder of *block 9* and the entirety of *block 10* (total = 27 noVF trials), with the other trials being VF trials (total = 54 trials). The experiment lasted 1 h. NoVF trials were used in manner consistent with previous studies (22, 31) to assess the size of the aftereffect in the two visual feedback groups (CVF vs. EPF).

Our methods differed in several ways from the typical approach use to study aftereffects. First, we included visual feedback trials in the aftereffect phase. This was done because piloting with the isometric force task indicated that long sequences of NoVF trials induced excessive variance. So, to stabilize this variance, we included VF trials. These VF trials could introduce some relearning of the baseline gain, potentially contaminating the aftereffect results. To ensure that such relearning, if it took place, did not differ across EPF and CVF groups, we directly compared VF performance across these groups during the aftereffect phase. Second, we did not explicitly instruct participants not to use a strategy during the aftereffect phase (i.e., exclusion trials). This was because we had not told participants to use a strategy at any time before the aftereffect phase, so asking them to stop using a strategy was not appropriate. Last, it should be noted that the gain learning was reversed in *experiment 2* relative to *experiment 1*, limiting direct comparisons between the two experiments. This was done to ensure that the aftereffect in *experiment 2* consisted of participants overshooting, rather than undershooting the target. Due to limitations of the equipment, large undershooting was undesirable, as small forces sometimes failed to trigger a detectable response above the background noise, which would have truncated the aftereffect and potentially introduced bias across groups.

### Analysis

#### Experiment 1.

For every trial, the time series of the force profile was low-pass filtered using a Butterworth filter (5 Hz) and the force velocity was calculated. In the CVF groups, response force for each trial was the point at which the force stopped increasing and stabilized, which was determined by taking the point at which the force velocity fell below 10% of the peak force velocity for that trial. This corresponded to what participants attended to on screen and were told would be used to judge their performance accuracy. In the EPF groups, the response force for each trial was the force at the point in time when the force velocity reached its peak, as this corresponded to the feedback presented on screen. In all groups, response force was multiplied by the visuomotor gain to transform the force to the visual metrics (i.e., bar height in different gain conditions). This value was transformed into an absolute difference from the target value (absolute error ratio) using the following equation;

$$\text{Absolute error ratio}=\left||\text{visual bar height}/\text{visual target height}-1\right||.$$

Here, an absolute error ratio of 0 indicates that the force produced was identical to the target level. For no visual feedback trials (noVF), to correct for force drifts before movement onset, the data were baseline corrected by subtracting the mean force level during the ready period from the final force level, before calculating the absolute error ratio. Unsmoothed absolute error ratio data are shown in Supplemental Fig. S1 (see https://doi.org/10.6084/m9.figshare.17030072.v1).

Transfer percentages, used to assess intermanual transfer of learning, were calculated from the absolute error ratios in the LnoVF and RnoVF conditions in the following manner for each participant. First, the intermanual error ratio was calculated using the absolute error ratio on LnoVF and RnoVF trials:

Intermanual error ratio=(LnoVF−RnoVF)/RnoVF.

Thus, if the absolute error ratio was equivalent for both hands, the intermanual error ratio would be 0, whereas if it was three times larger on the left hand, the intermanual error ratio would be 2. To make this value more intuitive, the transfer percentage was then generated by calculating the intermanual error ratio as a percentage of the maximum intermanual error ratio during the test phase, which was 2 (i.e., an intermanual error ratio of 2 is equivalent to an error 3 times larger on the left than the right hand, because none of the 3 times gain increase had been transferred).

$$\text{Transfer percentage}=\;\left(\left(2-\text{Intermanual error ratio}\right)/2\right)\;\text{×}\text{ 1}00$$

A transfer percentage of 100% therefore meant that the LnoVF absolute error ratio was the same as the RnoVF absolute error ratio, whereas a transfer percentage of 0% meant the LnoVF absolute error ratio was three times larger than the RnoVF absolute error ratio. It should be noted that by setting an upper limit on the error, we are simply normalizing to this upper limit. Participants were free to exceed this limit, meaning that transfer percentages can be below 0% or above 100%. The specific value used to define the maximum possible error does not change the results of the statistical tests and is used for display purposes. We used the value of 2 because this was the maximum expected error in the test phase (i.e., if no transfer occurred), and because it approximated the largest errors participants made during the practice session, before the baseline gain settings were learned.

Learning percentages for RVF and RnoVF trials were calculated separately in the same manner, in each case using the mean absolute error ratio at baseline and test.

Learning error ratio=(Test−Baseline)/Baseline

$$\text{Learning percentage}=\left(\left(2-\text{Learning error ratio}\right)/2\right)\;\text{×}\text{ 1}00$$

Group differences in transfer percentage at baseline and test, RVF learning percentage at test, and RnoVF learning percentage at test were all assessed using 2 × 2 between subject’s ANOVA, with factors of visual feedback type (CVF vs. EPF) and perturbation schedule (gradual vs. abrupt). We also calculated transfer and learning percentages throughout the experiment by applying the above formulae to every trial. These values were smoothed for display purposes via averaging within a five-trial moving window.

Reaction times (RT) were calculated for every trial by taking the point in time after the go signal where the force level rose above four times the standard deviation of the force during the ready period. Mean RT at baseline and test were compared across groups using a 2 × 2 × 2 mixed ANOVA, with the within subject’s factor of phase (baseline vs. test) and the between subject’s factors of visual feedback type and perturbation schedule. Trial level RT data were also smoothed for display purposes via averaging within a five-trial moving window.

Trials were automatically rejected from the analyses based on absolute error ratio if during the 1,500 ms go period, the participant failed to increase force above 10% of the target force level for that trial. We also rejected trials where peak force velocity occurred after the go period (i.e., late responses > 1,500 ms). Trials were rejected from the RT analyses if force increases were detected after the go period (late responses > 1,500 ms) or if RT was classified as being <100 ms. There were no significant differences between the CVF and EPF groups in terms of the mean percentage of trials rejected per participant from the error ratio analyses [Mn = 8.77%, SD = 4.96% vs. Mn = 11.58%, SD = 7.34%; *t*(54) = −1.649, *P* = 0.105] or the RT analyses [Mn = 15.28%, SD = 9.09% vs. Mn = 19.96%, SD = 12.44%; *t*(54) = −1.58, *P* = 0.12].

#### Experiment 2.

RT and absolute error ratio were calculated in the same manner as *experiment 1*, as were the learning percentages for RVF trials. We used the same trial exclusion criteria as *experiment 1*. There were no significant differences between the CVF and EPF groups in terms of the mean percentage of trials rejected in each participant from the error ratio analyses [Mn = 1.02%, SD = 0.64% vs. Mn = 0.81%, SD = 0.9%; *t*(27) = 0.685, *P* = 0.499] or the RT analyses [Mn = 10.94%, SD = 14.02% vs. Mn = 4.79%, SD = 6.99%; *t*(27) = 1.511, *P* = 0.142].

Unlike unperturbed reaching tasks, in our task there was no true baseline, as the force was always transformed onto a value shown on screen. It was therefore essential to establish that the baseline was long enough to serve as a “true” baseline. To check that the baseline phase was of adequate length, and that the initial gain had been learned (i.e., a baseline established), we compared absolute error ratio across the baseline period using a 2 × 2 mixed effects ANOVA, with the between subject’s factor of visual feedback type (CVF vs. EPF) and the within subject’s factor of baseline block (*block 1* vs. *block 2*).

To specifically assess the size of the aftereffect, we analyzed the signed error ratio (i.e., error ratio calculated without converting to absolute values).

Signed error ratio=(visual bar height/visual target height) − 1

Signed (as opposed to absolute) error ratio was used because during the aftereffect phase, the gain suddenly increased from 1 to 3 and we were interested in the degree to which participants overshot the target force level, as this would reflect the degree to which they had adapted to the lower gain setting during the learning and test phases. Using absolute error was not appropriate in this case because the direction of the errors needed to be preserved. We determined the size of the aftereffect for each participant for noVF trials by subtracting the mean signed error ratio during the baseline phase. The same approach was used for the VF trials during the aftereffect phase to check for evidence of different relearning rates across groups. To further check whether relearning influenced the results we conducted a 2 × 2 mixed effects ANOVA on the noVF data, with the between subject’s factor of visual feedback type (CVF vs. EPF) and the within subject’s factor of time (1st half vs. 2nd half of the noVF trials). For this test, a significant visual feedback type × time interaction could suggest differential influence of relearning across groups.

We compared mean RT throughout the entire experiment across the two visual feedback groups. We also specifically assessed whether the gradual gain change interacted with group by comparing RT change (test − baseline) in each visual feedback group and whether the sudden gain change before the aftereffect phase interacted with group by comparing RT change (aftereffect RVF RT − test RVF RT) in each visual feedback group. Independent samples *t* tests were conducted on all the variables of interest. Data were smoothed for presentation purposes in the same manner, as *experiment. 1*.

## RESULTS

### Reaction Times Were Slower for EPF Trials

**Figure 2.**
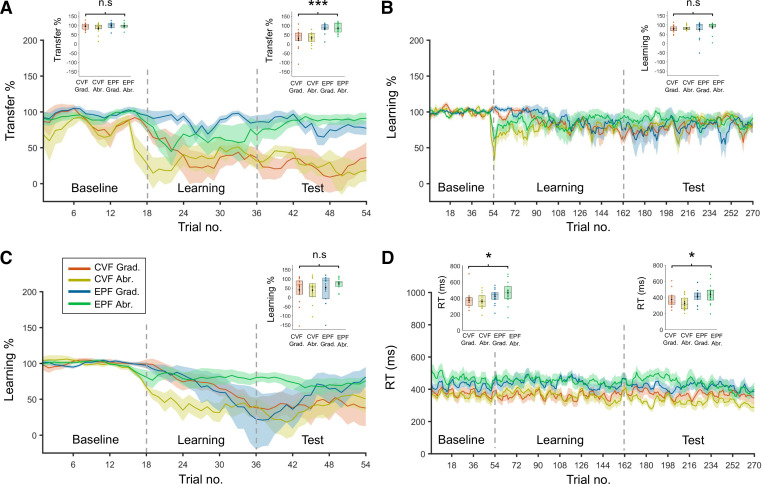
*Experiment 1*. Transfer, learning, and reaction time results. *A*: percentage transfer between RnoVF and LnoVF in each group across the entire experiment. In the endpoint feedback (EPF) groups the amount of transfer returned to around 85% during the learning phase, but in the continuous visual feedback (CVF) groups remained around 30% through to the end of the test phase. *Inset* box plots show that mean transfer percentage at baseline did not differ across groups but was significantly higher in the EPF groups than the CVF groups during the test phase (n.s., not significant, ***P < 0.001, *n* = 56). *B*: percentage learning of gain change relative to baseline performance on right hand feedback (RVF) trials in each group across the entire experiment. *Inset* box plot shows that learning rates did not significantly differ across groups during the test phase (n.s., not significant, *n* = 56). *C*: percentage learning of gain change relative to baseline performance on RnoVF trials in each group across the entire experiment. *Inset* box plot shows that the mean performance at test did not differ across groups (n.s., not significant, *n* = 56). *D*: response times on RVF trials for all groups across the entire experiment. RT was slower in EPF compared with CVF groups at baseline and test (*P < 0.05, *n* = 56). Abr., abrupt; Grad., gradual., LnoVF, left hand no feedback; RnoVF, right hand no feedback.

Cognitive strategy use has been associated with prolonged reaction times, possibly due to an increased planning load (24, 25). We found that RT for RVF trials was longer for the EPF groups than the CVF groups throughout the experiment (Fig. 2*D*). This manifested as a significant main effect of visual feedback type when baseline and test phases were analyzed for all four groups [*F*(1,52) = 7.041, *P* = 0.011]. There was a trend toward RT getting faster from baseline to test [main effect of phase: *F*(1,52) = 3.587, *P* = 0.064] but no significant interaction between visual feedback type and phase [*F*(1,52) = 0.032, *P* = 0.858]. There was no significant main effect of perturbation schedule [*F*(1,52) = 0.001, *P* = 0.975] and no significant interaction between visual feedback type and perturbation schedule [*F*(1,52) = 1.242, *P* = 0.270]. There was also no interaction between phase and perturbation schedule [*F*(1,52) = 1.742, *P* = 0.193] and no significant visual feedback type × perturbation schedule × phase interaction [*F*(1,52) = 0.013, *P* = 0.909].

When the baseline and test phases were analyzed separately, the same pattern of RT results was observed. There was a significant main effect of visual feedback type at baseline [*F*(1,52) = 5.761, *P* = 0.020], η^2^ = 0.1) and test [*F*(1,52) = 7.061, *P* = 0.010, η^2^ = 0.12]. Again, at baseline, there was no significant main effect of perturbation schedule [*F*(1,52) = 0.181, *P* = 0.673] or perturbation × visual feedback type interaction [*F*(1,52) = 0.993, *P* = 0.324), and at test there was no significant main effect of perturbation schedule [*F*(1,52) = 0.152, *P* = 0.699] or perturbation × visual feedback type interaction [*F*(1,52) = 1.274, *P* = 0.264].

Therefore, participants responded more slowly when EPF was available on visual feedback trials throughout the task, possibly by incorporating a cognitive strategy for movement planning. RT on these trials did not differ between abrupt and gradual groups.

### Visual Gain Change Was Successfully Learned for All Groups

To determine that the gain change was successfully learned by all participants, we calculated the learning percentage on RVF trials, which was the ratio between the absolute error ratio at a given point in time and the absolute error ratio at baseline, expressed as a percentage of the maximum error during the test phase (i.e., 100% = complete gain learning; 0% = gain not leaned, error ratio is 3 times larger than baseline error ratio). Learning percentages on RVF trials plateaued before the test phase in all groups (Fig. 2*B*) and were moderately high in all groups (∼80%). When learning percentages were directly compared at test, there was no significant main effect of visual feedback type [*F*(1,52) = 0.06, *P* = 0.807; Fig. 2*B* box plot]. There was also no main effect of perturbation schedule [*F*(1,52) = 0.786, *P* = 0.380] and no significant perturbation schedule × visual feedback type interaction [*F*(1,52) = 0.148, *P* = 0.720]. Thus, all groups showed comparable learning of the gain change.

### Right Hand No Visual Feedback Learning Did Not Differ across Groups

Learning percentages on RnoVF trials (Fig. 2*C*) were markedly worse than those seen for RVF, which was expected because visual feedback was not available to aid performance. Nevertheless, it was expected that RnoVF trials would show evidence of gain learning and that this learning should be comparable across groups. This was confirmed, with all groups showing ∼50% learning rates on RnoVF trials at test. Comparing across groups at test, there was no main effect of visual feedback type [*F*(1,52) = 1.764, *P* = 0.19; Fig. 2*C* box plot], no main effect of perturbation schedule [*F*(1,52) = 0.262, *P* = 0.611], and no significant perturbation schedule × visual feedback type interaction [*F*(1,52) = 0.376, *P* = 0.542]. So in summary, right hand performance, both for RVF and RnoVF trial types, did not significantly differ across groups.

### Greater Intermanual Transfer of Learning for Endpoint Feedback

Our main interest was whether a putative shift toward a cognitive strategy has any influence on the intermanual transfer of visuomotor adaptation. We calculated the transfer percentage, which was the ratio between the absolute error ratio of left and right no visual feedback trials, expressed as a percentage of the maximum error (to compare to group level unsmoothed absolute ratio for each arm individually, see Supplemental Fig. S1; see https://doi.org/10.6084/m9.figshare.17030072.v1). In the learning and test phase, only the right hand was exposed to the perturbation (i.e., visual feedback) but not the left. A transfer percentage close of 100% indicates comparable performance on both hands in the absence of visual feedback, i.e., that all learning on the right hand has been transferred to the left. A transfer percentage of 0% means no learning has been transferred (LnoVF error is 3 times larger than RnoVF), whereas 50% indicates half the learning has been transferred (LnoVF error is 2 times larger than RnoVF).

Transfer percentages at test in the EPF groups were higher than those in the CVF groups (EPF Grad. Mn = 84.64%, EPF Abr. Mn = 85.56% vs. CVF Grad. Mn = 30.5%, CVF Abr. Mn = 32.43%; Fig. 2*A* right box plot). ANOVA performed between groups revealed that there was a significant main effect of visual feedback group on the transfer percentage at test [*F*(1,52) = 31.194, *P* < 0.001, η^2^ = 0.37]. However, there was no significant main effect of perturbation schedule [*F*(1,52) = 0.022, *P* = 0.883] and no significant perturbation schedule × visual feedback type interaction [*F*(1,52) = 0.003, *P* = 0.958].

As can be seen from the trial level analysis (Fig. 2*A*), transfer percentages in the EPF groups during the learning phase showed some improvement until reaching a plateau around the start of the test phase. Conversely, transfer percentages in CVF groups were lower and plateaued earlier during the learning phase.

The transfer results were not due to baseline differences in left and right hand performance when the visual feedback was absent. Baseline transfer percentages were close to 100% in all groups. At baseline, there was no significant main effect of visual feedback type [*F*(1,52) = 2.95, *P* = 0.092; Fig. 2*A* *left* box plot], no significant main effect of perturbation schedule [*F*(1,52) = 1.665, *P* = 0.203], and no significant perturbation schedule × visual feedback type interaction [*F*(1,52) = 0.529, *P* = 0.471].

Thus, only the factor of visual feedback type influenced the transfer of learning from RnoVF to LnoVF trials, with EPF being associated with higher rates of intermanual transfer.

### Experiment 2 Results

Due to the short baseline period, it was necessary to ensure that performance had plateaued by the end of the practice session and did not continue to improve during the baseline phase. We compared the absolute error ratio in the first and second baseline blocks across visual feedback groups. There was no significant main effect of visual feedback type [*F*(1,27) = 0.884, *P* = 0.356], no significant main effect of block [*F*(1,27) = 0.008, *P* = 0.930], and no significant visual feedback type × block interaction [*F*(1,27) = 2.508, *P* = 0.125], indicating that performance was stable during the baseline phase and comparable across groups.

**Figure 3.**
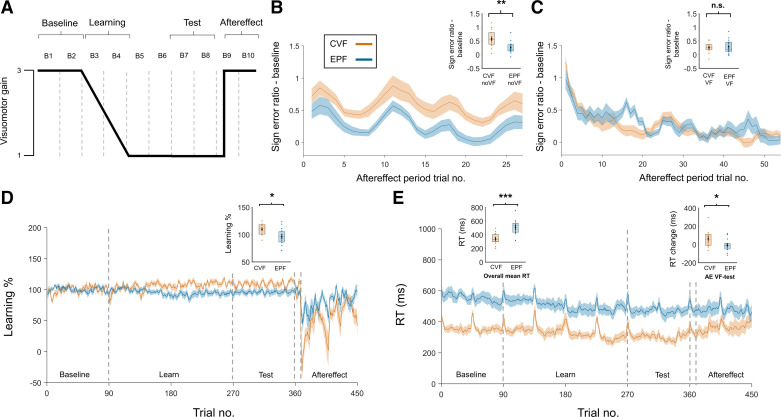
Design and results of *experiment 2*. *A*: design of *experiment 2* showing how visuomotor gain changed across blocks (*B1*–*B10*). On the 10th trial of *B9*, there was sudden gain change that returned the gain to the baseline level. During this aftereffect phase, trials alternated between two visual feedback (VF) trials followed by one no visual feedback (noVF) trial. *B*: signed error ratio after subtracting baseline values for noVF trials in the aftereffect phase, showing larger overshooting for continuous visual feedback (CVF) than endpoint feedback (EPF) groups, consistent with larger aftereffects. Box plot shows that the degree of overshoot was significantly higher for the CVF group relative to the EPF group, indicative of a greater aftereffect (**P < 0.01, *n* = 29). *C*: signed error ratio after subtracting baseline values for visual feedback (VF) trials in the aftereffect phase for CVF and EPF groups. *Inset* shows that there was no significant difference between the two groups (n.s., not significant, *n* = 29). *D*: right hand feedback (RVF) learning percentage across entire experiment for CVF and EPF groups. Note that both groups were able to maintain performance accuracy close to 100% from the baseline to the test phase. *Inset* shows that learning percentage at test was significantly higher in the CVF group compared with the EPF group (*P < 0.05, *n* = 29). *E*: mean reaction time (RT) across entire experiment for CVF and EPF groups. Left box plot shows that overall RT was significantly slower in the EPF group compared with the CVF group. Right box plot shows that the CVF group increased their RT on VF trials from the test phase to the aftereffect phase to a greater extent than the EPF group (*P < 0.05, ***P < 0.001, *n* = 29).

The purpose of *experiment 2* was to determine whether EPF was associated with smaller aftereffects than CVF, as smaller aftereffects have been associated with strategy use (23, 25, 27). This was tested by assessing noVF trials after a sudden increase in visuomotor gain, which raised the gain back to baseline levels, following an extended period of adaptation to a gradually introduced lower level of visuomotor gain (Fig. 3, *A* and *B*). On noVF trials, the degree of overshoot was significantly larger for the CVF compared with the EPF group [Mn = 0.58, SD = 0.31 vs. Mn = 0.26, SD = 0.21; *t*(27) = 3.247, *P* = 0.003, Cohen’s d = 1.2], indicating a larger aftereffect was likely present in the CVF group.

VF trials were included to help stabilize large variances in the data that arose if only noVF trials were included. To assess whether these VF trials induced different relearning of the gain across different feedback groups, which could have influenced the aftereffect results, we compared VF performance, finding that there were no significant differences between the CVF and EPF groups (Mn = 0.26, SD = 0.18 vs. Mn = 0.29, SD = 0.25; *t*(27) = −0.395, *P* = 0.696; Fig. 3*C*). We also checked whether performance on noVF trials changed over the course of the aftereffect phase. As already reported, there was a significant main effect of visual feedback type [*F*(1,27) = 10.583, *P* = 0.003, η^2^ = 0.282]. Unsurprisingly, because the aftereffect necessarily decreases, there was also a significant main effect of time [*F*(1,27) = 22.997, *P* < 0.001, η^2^ = 0.46]. If different relearning across groups had strongly influenced the NoVF results, then we would have observed a significant visual feedback type × time interaction. Crucially, however, this was not the case [*F*(1,27) = 0.250, *P* = 0.621], meaning that the rate of aftereffect reduction likely did not change differently across groups. So overall, we found no evidence to suggest that the aftereffect result could be explained by group differences in relearning.

We also examined how well the initial gradual gain decrease was learned. The learning percentage, based on the absolute error ratio, was close to 100% for both groups (Fig. 3*D*), but was significantly higher in the CVF group compared with the EP group [CVF Mn = 109.24%, SD = 10.09% vs. EPF Mn = 95.97%, SD = 15.44%; *t*(27) = 2.719, *P* = 0.011, Cohen’s *d* = 1.02]. Thus both groups learned the gradual gain change, but participants in the CVF group actually performed slightly better at test than baseline, whereas those in the EPF group performed slightly worse at test than baseline.

We replicated the finding from *experiment 1* that EPF was associated with longer RT than CVF [Mn = 506.14 ms, SD = 113 ms vs. Mn = 340.25 ms, SD = 81.3 ms; *t*(27) = 4.509, *P* < 0.001, Cohen’s *d* = 1.69; Fig. 3*E* *left* box plot], again consistent with the hypothesis that EPF promotes strategy use. As with *experiment 1*, there was a general tendency for participants to respond faster from baseline to test, but this speeding of responses did not differ between CVF and EPF groups [Mn = −53.08 ms, SD = 53.98 ms vs. Mn = −83.67 ms, SD = 41.73 ms; *t*(27) = 1.714, *P* = 0.098]. This RT effect was more pronounced than *experiment 1*, which may have been due to the simplicity of the task (i.e., no hand switching) or the different visuomotor gain change pattern used in the two experiments.

Interestingly, participants in the CVF group tended to increase RT after the sudden gain change (aftereffect phase) on VF trials, while those in the EPF groups maintained their RT [Mn = 61.95 ms, SD = 90.93 ms vs. Mn = −7.68 ms, SD = 65.90 ms; *t*(27) = 2.373, *P* = 0.025, Cohen’s *d* = 0.88; Fig. 3*E* *right* box plot]. Indeed, by the end of the aftereffect phase, group mean CVF RT rose to be similar to EPF RT. These results might indicate that the sudden gain change resulted in a greater reliance on strategy use in the CVF group. This RT difference did not appear to improve performance selectively in the CVF group, as evidenced by the VF trial results (see Fig. 3*C*). If it did have some small effect, it would have served only to reduce our effect of interest (i.e., the aftereffect amplitude).

When questioned at the end of the experiment, all participants in both groups reported being aware of the sudden gain increase (aftereffect phase), while remaining unaware of the earlier gradual gain decrease.

## DISCUSSION

Encountering a change in the environment, humans can maintain their motor performance by either adapting their sensorimotor representation or by using a cognitive strategy to compensate for the change (2, 26). We examined whether elements of the design of a visuomotor task can facilitate a cognitive strategy and whether this in turn enhances the intermanual transfer of learning. Across two experiments, when the visual feedback of our ballistic force production task was restricted to the end point, reaction times increased, suggesting a greater reliance on a cognitive strategy to solve the task (25, 32). Following this pattern, intermanual transfer was facilitated in the endpoint feedback condition, indicating that a cognitive strategy may facilitate effector-independent learning. We observed additional evidence that EPF promoted strategy use via a second experiment, which generated results consistent with reduced aftereffects for EPF relative to CVF.

Restricting visual feedback to the end point of the task (EPF) has been shown to facilitate cognitive control (20, 22). During prism adaptation, EPF may enhance the generalization of learning across effectors (33) and intermanual transfer (34), because it promotes greater strategic control than continuous visual feedback. Our results support the view that EPF encourages strategic learning because it involves a single performance error signal pertaining to goal completion. It therefore encourages learning at the planning stage, above the level of the control policy (35). Strategic learning and motor adaptation have been suggested to involve dissociable brain networks (5). The supplementary motor area is central to strategic control and bimanual tasks (4), making it a likely candidate region for the processing of EPF and the associated generalizable learning we observed.

Cognitive strategies have always been considered more time consuming than motor adaptation (36). During an isometric visual rotation task, EPF was associated with slower RT, and the introduction of perturbations selectively slowed responses further under conditions of EPF (20). Other studies have observed a relationship between cognitive strategy and longer RT (23, 24, 37). Our results suggest that longer responding in our task can indicate strategic control throughout the task and that this form of learning can enhance performance when flexible responding is required.

In our task, RT was not manipulated independently of visual feedback type, meaning that other interpretations of the prolonged RT in the EPF group, such as task difficulty, could not be completely excluded. It was therefore necessary to verify that EPF did indeed promote strategy use. In a second experiment we found that having learned a gradual decrease of visuomotor gain, participants tended to overshoot the target after the gain suddenly increased back to baseline levels. These apparent aftereffects in response to the “switching off” of a perturbation were broadly consistent with those seen during reaching tasks (9, 38–40). We found evidence that aftereffect amplitude was reduced in the EPF group relative to the CVF group, consistent with previous reports finding reduced aftereffects for EPF (20, 21). Aftereffects are a hallmark of motor adaptation and reductions in aftereffects have previously been found to be caused by the use of cognitive strategies (23, 25, 27).

However, two important caveats must be noted relating to the design of our second experiment. First, due to constraints of the equipment, the direction of the gain change in *experiment 2* was reversed relative to *experiment 1*, limiting direct comparisons of the data. Second, VF trials were included in the aftereffect phase, potentially introducing relearning of the gain, which could have influenced the aftereffect results, particularly if relearning rates differed across groups. To mitigate this latter issue, we analyzed the VF trials across groups and checked for changes in NoVF over time. In both cases, we did not find any evidence that relearning was different in the two groups. Overall, our results were therefore consistent with EPF being associated with reduced aftereffects, providing additional evidence that the enhanced intermanual transfer of learning associated with EPF can be ascribed to greater strategy use.

Changing the perturbation schedule from gradual to abrupt did not increase the RT, an indicator of strategy use, and consequently did not facilitate intermanual transfer of learning. Previous work indicated that abrupt gain changes enhance intermanual transfer of learning via the facilitation of cognitive strategies (15, 16) but opposite results also exist (17–19). As this strategy use is assumed to be due to the abrupt change causing the perturbation to reach explicit “awareness” (16, 41), difficulty in setting the size of the abrupt change and interindividual variability in change sensitivity may explain the null effect in the present study. In *experiment 1* abrupt gain changes were possibly also less salient than normal due to the constant switching between response hands. Indeed, in *experiment 2*, we observed some evidence that an abrupt change in gain (aftereffect phase) could produce some gradual slowing of RT in the CVF group, consistent with a slight increase in strategy use at the end of the task.

We found transfer percentages of ∼85% for EPF, whereas previous motor tasks report ∼25% (18, 42–44). The disparity may be because of greater strategy use in our task, but it is also likely because transfer was assessed continuously, which has been shown to increase transfer rates from the 25% seen in blocked designs to above 50% (17). In addition, we used transfer from RnoVF to LnoVF trials, which controlled for task difficulty across conditions, but inherently gives larger transfer values than comparing to visual feedback trials. Caution is therefore required when comparing transfer rates across paradigms. We also only tested transfer from the right to the left hand. Several studies have reported that transfer is reduced from the nondominant to the dominant arm (45, 46), whereas others have found no such asymmetry (47–49). Future work is needed to address how left to right transfer works during force production tasks.

Motor learning occurs at multiple levels of the control hierarchy (50), with movement planning involving effector-dependent and effector-independent brain regions (51). Intermanual transfer of learning has generally been assumed to be achieved by updating such effector-independent motor representations (14). However, an effector-independent cognitive control strategy, such as reaiming (23), can achieve the same result. Future models of intermanual transfer need to consider the role of cognitive strategies.

In conclusion, our results add to the growing body of literature showing that elements of the task environment, such as the type of visual feedback available, can alter the balance between cognitive strategies and motor adaptation. The involvement of a cognitive strategy likely enhances intermanual transfer of learning. This greater generalization may result from strategic learning being related to movement planning, and as such being located above the control policy in the motor hierarchy.

## SUPPLEMENTAL DATA

10.6084/m9.figshare.17030072.v1 Fig. S1: https://doi.org/10.6084/m9.figshare.17030072.v1

## GRANTS

J.D.H. was supported by a UCL-NTT Impact studentship and by a NICT Internship Trainee Program. N.H. is supported by Japan Society for the Promotion of Science (Kakenhi 26119535, 18H01106). Y.I. and N.H. are supported by ERATO (JPMJER1801).

## DISCLOSURES

No conflicts of interest, financial or otherwise, are declared by the authors.

## AUTHOR CONTRIBUTIONS

J.D.H., P.H., H.G., S.B., Y.I., and N.H. conceived and designed research; J.D.H. performed experiments; J.D.H. analyzed data; J.D.H. and N.H. interpreted results of experiments; J.D.H. prepared figures; J.D.H. drafted manuscript; J.D.H., P.H., H.G., S.B., Y.I., and N.H. edited and revised manuscript; J.D.H., P.H., H.G., S.B., Y.I., and N.H. approved final version of manuscript.
